# Comment on “Dental Prosthetic Status and Prosthetic Need of the Institutionalized Elderly Living in Geriatric Homes in Mangalore: A Pilot Study”

**DOI:** 10.1155/2013/535480

**Published:** 2013-05-02

**Authors:** Ashutosh Dixit, Varun Arora, Kapil Loomba, Ridhima Birmani Gaunkar, Seema K. Dixit, Bhaskar Agarwal, Alok Misra, Narendra Kumar Gupta

**Affiliations:** ^1^Department of Periodontics, Seema Dental College, Rishikesh 249203, India; ^2^Active Research Group, Arun Professional Services, Tulsidas Marg, Chowk, Lucknow 226003, India; ^3^Department of Conservative Dentistry, Saraswati Dental College, Lucknow 227105, India; ^4^Department of Public Health Dentistry, Goa Dental College, Goa 403202, India; ^5^Department of Endodontics & Conservative Dentistry, Seema Dental College, Rishikesh 249203, India; ^6^Department of Prosthodontics, Faculty of Dental Sciences, King George Medical University, Lucknow 226003, India; ^7^Department of Endodontics & Conservative Dentistry, Career Institute of Dental Sciences and Hospital, Lucknow 226002, India; ^8^Department of Prosthodontics, Babu Banarasi Das College of Dentistry, BBD University, Lucknow 227015, India

## Abstract

Public Health Dentistry is a speciality which is targeted towards the larger benefit of community and society. Dental health surveys in specific population groups should be planned adequately and the data should be analyzed in such a way so that it may help in making strategies for the intervention to improve the existing status. This could be only done with the help of proper planning, analysis and interpretation of a sample survey. The present study highlights the research design, statistical and inferential errors in a published work of public health dentistry in order to bring about the common mistakes and errors made. The renewed suggested approach helps in interpreting the results in a better way and makes them objective-oriented.

This is in reference to the research article entitled, “*Dental Prosthetic Status and Prosthetic Need of the Institutionalized Elderly Living in Geriatric Homes in Mangalore: A Pilot Study*” published in ISRN Dentistry [1] regarding which we find the following discrepancies in the paper and request publication of an erratum for the benefit of the scientific community.

*Comment  1*. In Abstract section, under Results, the authors state “*Eighty-eight percent of those examined were fully edentulous*,” however, in Table  3 full-prosthesis need has been shown as 46.6% and 41.4% for upper and lower arches, respectively. As there is a substantial difference in authors' statement “*Eighty-eight percent of those examined were fully edentulous*” and the full-prosthesis need shown in Table  3 to be 46.6% and 41.4% for upper and lower arches, respectively. In fact the figure of “Eight-eight percent” seems to be the finding of “prosthetic status” (Table  2; for individuals having no prosthesis) [1]. 

*Comment  2*. In Results section (paragraph 2, last three lines) authors state “*significant differences between the genders and the prosthetic status of their upper arches ( χ* ^2^ = 5.105*; P* = 0.024*) and lower arches ( χ* ^2^ = 5.105*; P* = 0.024)” which is a misrepresentation of the finding which shows “*significant differences between the genders for prosthetic status of their upper arches ( χ* ^2^ = 5.105*; P* = 0.024*) and lower arches ( χ* ^2^ = 5.105*; P* = 0.024)” [1]. 

*Comment  3*. In Discussion (paragraph 2), the authors mention that “*Another finding was that the prosthetic status was slightly better in males than in females. Females usually depend on male members of their families to take them for treatment*” [1]—it seems to us that the authors have ignored some vital statistics already shown in their paper while making this inference. This can be illustrated by the findings in the paper itself, as Table  1 shows majority of females to be within 70 years of age (38/60; 63.3%) which is significantly higher as compared to males (32/73; 43.8%) (*P* = 0.025), thus it is misleading to conclude that the prosthetic status of females was slightly better in males than in females. It might be possible that females have lower prosthetic need owing to females being younger in age as compared to males. This assumption of ours gets impetus with the fact that the gender-wise prosthetic need assessment (Table  3) shows the proportion of males with need for full prosthesis to be higher as compared to females (50.7% versus 41.7% for upper arch and 43.8% versus 38.3% for lower arch). If we take into account those cases in whom the prosthetic need was not recorded then this figure changes (53.6% versus 41.7% for upper arch and 46.4% versus 38.3% for lower arch). It is pertinent to mention here that the issue of age of 70 has been considered because in the given class intervals it is the median age (Table  1; 70/133 subjects are aged <70 years and 63/133 subjects are aged above 70 years). The authors must know that they can draw conclusions such as “the prosthetic status was slightly better in males than in females” only after proving that except for gender both the groups were matched demographically. Unfortunately, authors have not carried out any statistical analysis for Table  1 for which we calculated the difference in age between two genders to be significant statistically (*χ* ^2^ = 59.7; *P* < 0.001). After employing this statistical tool, age emerges as confounder. The table also shows that need for one unit prosthesis and multiunit prosthesis was higher among females, itself is an indicator to understand that if one-unit and multiunit prosthesis need as against combination of one-unit and multiunit and full prosthesis is higher among females then prosthetic status of males is better (as combination of one-unit and multiunit and full prosthesis is undoubtedly an indicator of prosthetic need of “higher rung” vis-à-vis worse prosthetic indicator as compared to those who require only “one-unit” and “multiunit” prosthesis). The authors' conclusions in this regard seem to be biased on their presumptive assumptions only and are not substantiated statistically. 

*Comment  4*. It is disturbing to see the method of calculation of prosthetic need assessment. The authors have shown a total of 13 males and 3 females to be full prosthesis wearers (Table  2); however, the prosthetic need assessment has been done in entire 133 subjects. In our opinion, in such a case, a proper mention of unmet prosthetic needs (needs existing—needs fulfilled) should have also been made in order to elucidate the findings clearly as concluded arbitrarily by the authors (*Conclusions section*—*“The findings of this study clearly demonstrate a high unmet need for prosthetic care among the institutionalized elderly population surveyed*)” [1]. 

*Comment  5*. We are sorry to state that despite the evidence enumerated for gender-wise differences (*as enumerated by the authors*), the conclusion section does not include them at all. It is amusing to see that the authors, despite displaying a gender-wise comparison in all the three illustrations (Tables  1, 2, and 3) do not consider that gender-wise differences to be the main findings of the study so as to worth mention in conclusion section. It is defying the objectives of the study itself which state “*In order…evaluate oral health services*” [1] and holds relevance of citation of age wise and gender-wise differences in prosthetic status and unmet prosthetic needs in order to formulate appropriate “policies” for targeted and demographically differentiated groups.

Thus the findings of the study should be viewed with changed prosthetic status ad prosthetic need of the sampled population which is much lower than the 88% reported in the paper (actual figures need to be verified by the authors), existence of significant differences between the genders for prosthetic status of their upper arches and lower arches, lower age-adjusted prosthetic status and higher order of prosthetic need among females, and inappropriateness in mentioning the unmet prosthetic need of the sampled population.
